# The Formation of Fruit Quality in *Cucumis sativus* L.

**DOI:** 10.3389/fpls.2021.729448

**Published:** 2021-09-23

**Authors:** Juping Zhang, Shengjun Feng, Jing Yuan, Chen Wang, Tao Lu, Huasen Wang, Chao Yu

**Affiliations:** ^1^Collaborative Innovation Center for Efficient and Green Production of Agriculture in Mountainous Areas, College of Horticulture Science, Zhejiang A&F University, Hangzhou, China; ^2^State Key Laboratory of Subtropical Silviculture, Laboratory of Plant Molecular and Developmental Biology, College of Forestry and Biotechnology, Zhejiang A&F University, Hangzhou, China; ^3^Institute of Vegetables and Flowers, Chinese Academy of Agricultural Sciences, Beijing, China

**Keywords:** cucumber, fruit quality, QTLs, spine, bitterness, grafting

## Abstract

Cucumber is one of the most widely grown vegetables in China and an indispensable fresh fruit in the diet. With the development of society, the demand of people for cucumber quality is higher and higher. Therefore, cultivating high-quality cucumber varieties is one of the main goals of cucumber breeding. With the rapid development of biotechnology such as molecular marker, cucumber quality control network is becoming clear. In this review, we describe the formation mechanism of cucumber fruit quality from three aspects: (1) the commercial quality of cucumber fruit, (2) nutritional quality formation, and (3) flavor quality of cucumber fruit. In addition, the determinants of cucumber fruit quality were summarized from two aspects of genetic regulation and cultivation methods in order to provide ideas for cucumber researchers and cultivators to improve fruit quality.

## Introduction

Cucumber (*Cucumis sativus* L.) is an annual herbaceous climbing fruit vegetable that belongs to the Cucurbitaceae family and originates from the tropical rainforest in the southern foot of the Himalayas. According to its geographical locations, it can be categorized into four groups such as the Indian group, the Eurasian group, the East Asian group, and the Xishuangbanna group (Qi et al., 2013). In China, cucumber can be subdivided into two geographic groups, such as the northern China group with dark green, dense spines and warts on fruits, and the southern China group with light green, sparse spines and warts on fruits (Jiang et al., 2015). Cucumber is an economically important crop in the world. Its fruits are fragrant and delicious with nutrient enrichment that can be consumed in fresh or processed into pickles. In addition, its fruits are also used in beauty products. However, with the constantly change in the cultivation environment and techniques, and the ever-rising living standards of people, the cucumber fruit quality is becoming much more concerned by the consumers.

With the continuous work on the formation of cucumber fruit quality by various research groups in recent years, some of the influencing factors and regulation mechanism of the formation of cucumber fruit quality can be understood now in a more in-depth and thorough manner. This review attempted to summarize the recent advances in the studies on the formation of cucumber fruit quality through physiological and molecular biological approaches in order to provide insights for further research studies on the formation of fruit quality of cucumber and other melons.

Fruit quality in cucumber can be defined by three aspects, namely, commercial quality, nutrient quality, and flavor quality (Lv et al., 1992). The commercial quality in cucumber contains the fruit size and shape, fruit spine characteristics (color, size, and density), fruit skin characteristics (color, ridges, and speckles), and flesh characteristics (color) (Wang et al., 2007; Lu et al., 2015; Zhang et al., 2016; Zhang C. et al., 2019). Fruit size and shape are the two most obvious appearances of quality traits in cucumber, which had become one of the criterions for breeders to select high fruit quality cultivars. The external fruit qualities, such as the fruit skin and spine color, the presence or absence of the wax on the cucumber surface, and the number, shape, size, distribution, and density of fruit spines (trichomes on cucumber fruit are called spines), are also important fruit quality traits for cucumber production (Choi et al., 2013; Li et al., 2013; Chen et al., 2014; Liu X. et al., 2016). In the late 1980s, the bloomless cucumber fruits are popular in Japan due to their distinct shiny appearance (Ohara et al., 2006). Besides, the character of spines could also have a big influence on consumer preference. For example, people in Europe prefer cucumber without spines, whereas people in Asia like cucumber with spines (Chen et al., 2014). Furthermore, epicuticular wax, which acts as the outermost barrier between the plants and their environment, is also one of the significant commercial quality traits in cucumber that can play significant roles in protecting the tissues against various biotic and abiotic stress (Samuels et al., 2008). Moreover, fruit flesh color that varies from white to green or yellow to orange is also an important fruit quality trait that influences the preference of consumers (Cuevas et al., 2010).

On the other hand, nutrition elements, such as soluble solids, vitamins, and minerals, constitute the nutrient quality in cucumber. In addition, flavor quality in cucumber, for one reason, contains all of the volatile compounds from cucumber, and for another reason, it is related to the non-volatile flavor substances (Kemp et al., 1974; Malundo et al., 1995). Ever since the 1960s, there have had research studies on the flavor of cucumber (Forss et al., 1962). Nowadays, there are more and more research studies on fruit flavor, which would help us to improve the fruit quality of cucumber.

## Genetic Regulation of Fruit Quality in Cucumber

### Genetic Regulation of Commercial Quality in Cucumber

**FIGURE 1:**
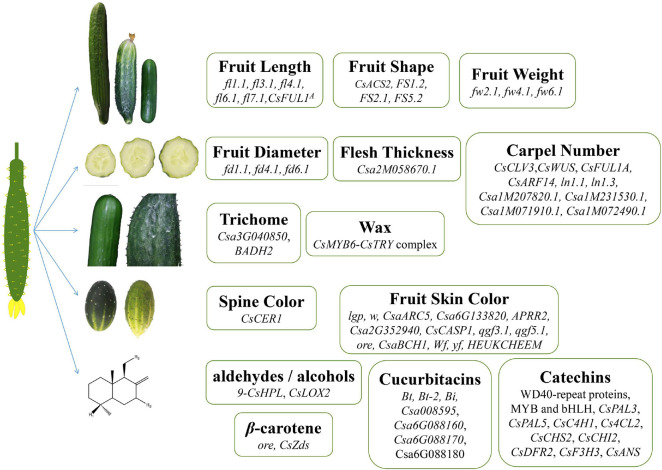
Genes related to cucumber fruit quality. Genes regulating fruit length, fruit shape, weight, diameter, thickness, carpel number, trichome, wax, spine color, and synthesis of secondary metabolite in cucumber.

Cucumber commercial quality is determined mainly by its fruit-related traits. The genes related to the commercial quality traits of cucumber fruits are shown in Figure 1 and Table 1. The ability of cucumber to produce large fruits is believed to have been evolved through long time domestication of wild cucumber, and *Cucumis sativus* L. var. hardwickii is regarded as the ancestor of cultivated cucumber. Its fruit is small and round and about 3–5 cm long. In addition, it has large amounts of spines on its epidermis. It is so bitter that it is difficult to eat (Sebastian et al., 2010; Yang et al., 2012). Nowadays, the fruit length of widely cultivated cucumber is about 10–30 cm, and the fruit shape has also become oblong, which has shown great variation compared with its ancestors. Besides, based on the whole genome sequencing and the construction of the high-resolution genetic map, we have gained a clearer understanding of the genetic mechanism of formation of fruit quality during their domestication processes (Huang et al., 2009; Qi et al., 2013).

**TABLE 1:** Genes related to the commercial quality of cucumber fruits.

**Category**	**Traits**	**QTLs or Gene name**	**References**
Commercial quality	Fruit length	*fl1.1, fl3.1, fl4.1, fl6.1*, and *fl7.1*	Bo et al., 2015
		*CsFUL1* ^*A*^	Zhao et al., 2019
	Fruit diameter	*fd1.1, fd4.1*, and *fd6.1*	Bo et al., 2015
	Fruit weight	*fw2.1, fw4.1*, and *fw6.1*	Bo et al., 2015
	Fruit shape	*CsACS2*	Tan et al., 2015
		*FS1.2*, *FS2.1*, and *FS5.2*	Pan et al., 2017
	Carpel number	*CsCLV3, CsWUS, CsFUL1^*A*^*, and *CsARF14*	Che et al., 2020
		*ln1.1, ln1.3, Csa1M207820.1, Csa1M231530.1, Csa1M071910.1*, and *Csa1M072490.1*	Zhang et al., 2015
	Flesh thickness	*Csa2M058670.1*	Xu et al., 2015
	Fruit skin color	*lgp*	Wehner et al., 2001
		*w*	Dong et al., 2012
		*CsaARC5*	Zhou et al., 2015
		*Csa6G133820*	Lun et al., 2016
		*APRR2*	Liu H. et al., 2016
		*Csa2G352940* and *CsCASP1*	Hao et al., 2018
		*qgf3.1* and *qgf5.1*	Bo et al., 2019
		*ore*	Bo et al., 2012
		*CsaBCH1*	Qi et al., 2013
		*wf* and *yf*	Kooistra, 1971; Whalen, 2005; Lu et al., 2015
		*HEUKCHEEM*	Zhang C. et al., 2019
	Spine color	*CsCER1*	Wang et al., 2015
	Wax	*CsMYB6*-*CsTRY* complex	Yang et al., 2018
	Trichome	*Csa3G040850*	Wei et al., 2016
		*BADH2*	Yundaeng et al., 2015

#### The Size and Shape of Cucumber Fruits

Fruit size and shape, especially fruit length, are important fruit traits during cucumber domestication and diversifying selection. Wenzel et al. (1995) first identified the overall information of gene regulation on the cucumber fruit length, diameter, and other genetic traits. Yuan et al. (2008) constructed a 257-point genetic linkage map and found 78 quantitative trait locus (QTLs) related to fruit weight, fruit length, fruit diameter, and other four fruit-related traits, which can be used to conduct marker-assisted selection in cucumber breeding. Bo et al., also detected several QTLs of cucumber fruit-related traits, including five fruit length-related QTLs (*fl1.1, fl3.1, fl4.1, fl6.1*, and *fl7.1*), three fruit diameter-related QTLs (*fd1.1, fd4.1*, and *fd6.1*), and three fruit weight-related QTLs (*fw2.1, fw4.1*, and *fw6.1*). At the same time, they also found that the chromosomal rearrangements of cucumber ancestors between wild and cultivated cucumbers were mainly concentrated on chromosomes 4, 5, and 7 (Bo et al., 2015). Besides, Cheng et al. (2010) and Wang et al. (2014) also found 5 fruit length (Fl)-related QTLs that were distributed in chromosome 1, 4, and 6 and 4 stalk length (Fsl)-related QTLs that were located on chromosome 3, 4, and 6, respectively.

The enzyme 1-aminocyclopropane-1-carboxylic acid synthase (ACS) in the process of plant hormone ethylene synthesis plays an important role in cucumber sex determination. The genes involved in this process include *CsACS1G* (Kamachi et al., 1997), *CsACS2* (Knopf and Trebitsh, 2006; Li et al., 2009), and *CsACS11* (Boualem et al., 2015). Among them, the *CsACS2* mutation makes cucumber produce a bisexual flower phenotype. *CsACS2* gene was eliminated in addition to the above functions, and it is also related to the length of the cucumber fruit. Boualem et al. (2009) proved that an allele of *CsACS2* co-segregated with the M (andromonoecious) locus, resulting in a round fruit phenotype after the gene mutation. Subsequently, Tan et al. (2015) further studied through fine mapping and found that another allele mutation of *CsACS2* caused the cucumber to appear a long fruit phenotype. Later, Pan et al. (2017) found that round fruit shape in WI7239 cucumber was controlled by two interacting quantitative trait loci, such as *FS1.2* and *FS2.1*, and demonstrated that *FS2.1* may encode a homolog of tomato fruit shape gene *SUN*. They also identified a *FS5.2* QTL in Xishuangbanna cucumber that has great significance on round fruit determination (Pan et al., 2017). Besides, Zhao et al. (2019) further demonstrated that among the QTLs that have putative functions in regulating cucumber fruit length, a gain-of-function allele *CsFUL1*^*A*^ can prevent the accumulation of auxin by inhibiting the expression of its transporters *PIN-FORMED1* (*PIN1*) and *PIN7*. This further analyzes the molecular mechanism of auxin regulating cucumber fruit development.

#### The Carpel Number and Flesh Thickness of Cucumber Fruits

In addition, factors that affect important fruit characteristics such as cucumber fruit shape, size, and internal quality also include the carpel number and the thickness of fruit flesh. The expression of *CsCLV3* in cucumber was negatively correlated with the number of carpels. *CsCLV3* and *CsWUS* act as negative regulators and positive regulators of changes in carpel number, respectively, and *CsWUS* can be directly combined with the promoter of *CsCLV3* to activate its expression. *CsFUL1*^*A*^ overexpression plants showed increased petals and carpels. Through the interaction of *CsARF14* and *CsWUS*, auxin can also participate in the change of cucumber carpel number (Che et al., 2020). The QTL mapping of Xishuangbanna cucumber revealed that *ln1.1* and *ln1.3* located on chromosome 1 are the main QTLs controlling multi-ventricular traits. At the same time, two genes, namely, *Csa1M207820.1* and *Csa1M231530.1*, involved in plant hormone signal transduction and two genes, namely, *Csa1M071910.1* and *Csa1M072490.1*, related to WD40 repeat protein are predicted as candidate genes (Zhang et al., 2015). By combining the separation and segmentation analysis with the sequencing of amplified fragments of specific lengths, the genes that regulate the pulp thickness of cucumber fruit were finely mapped, and the quantitative trait locus that controls the pulp thickness was located in the interval of about 0.19 Mb on chromosome 2. This 0.19-Mb region predicts and recognizes 20 genes, among which there is a 4 bp deletion mutation in the promoter region of the candidate gene *Csa2M058670.1* (a protein-lysine methyltransferase, PKMT), which may lead to the loss of its activity in the thin fruit line. This suggests that *Csa2M058670.1* may be a candidate gene for controlling cucumber pulp thickness (Xu et al., 2015). *Csa2M058670.1* belongs to the same subfamily as At2g18850, and the latter is related to the cell division and growth process of *Arabidopsis* (Horvath et al., 2003). At present, there are too many factors affecting the thickness of pulp and the number of ventricles in cucumber, and these two traits are easily affected by the environment, so a consistent conclusion has not yet been reached.

#### The Color of the Flesh and Skin of Cucumber Fruit

The colors of the fruit flesh and skin are also significant commercial quality traits in cucumber that have obvious influences on the choice of the consumers. Fruit color was determined by the regulation of pigment in the plants, and chlorophylls were declared to be the main factor to determine the fruit skin color (Egea et al., 2010). In cucumber, five genes controlled fruit skin color, such as dark green (*DG*), green (*dg*), yellow green (*yg*), light green (*lgp*), and white (*w*) peeling genes (Pierce and Wehner, 1990; Dong et al., 2012). Now, the fruit light green peel gene *lgp* and the white peel gene *w* have been identified (Wehner et al., 2001; Dong et al., 2012). The mutation of *CsaARC5* (*ACCUMULATION AND REPLICATION OF CHLOROPLASTS 5*), the ortholog gene of *Arabidopsis ARC5*, led to a light green fruit peel phenotype in cucumber (Zhou et al., 2015). Another Ycf54-like protein-encoding gene *Csa6G133820* can also determine the formation of light green fruits (Lun et al., 2016). Besides, a single-nucleotide insertion on *APRR2* disturbed the chlorophyll accumulation and chloroplast development so that leading to a white fruit color in cucumber (Liu H. et al., 2016). Identification of cucumber yellow green peel-related genes and research studies on their regulation mechanism have also progressed greatly in recent years. Research studies on a cucumber yellow green peel mutant (*ygp*) identified a *Csa2G352940* gene, encoding a MYB36 transcription factor, functioned to regulate a yellow green peel determination in cucumber. This study also revealed that *CsMYB36* may interact with the peel color development-related genes, such as Casparian strip (*CsCASP1*) and pigment synthesis protein (*CsMYC2*), to regulate yellow green peel determination in cucumber (Hao et al., 2018).

Besides fruit skin color, chlorophylls can also influence the formation of flesh color. Bo et al. (2019) revealed that two QTLs, *qgf3.1* and *qgf5.1*, can function together in regulating the formation of green fruit flesh in cucumber. When cucumber fruits developed to the mature stage, their flesh color changed from green to yellow or green to orange. High β-carotene content was demonstrated to be the main reason for cucumber to form fruits with orange flesh color, and further genetic analysis demonstrated that the quantity of β-carotene was controlled by the orange endocarp (*ore*) gene (Bo et al., 2012) and *CsaBCH1* (Qi et al., 2013). Cucumber fruits with white flesh and yellow flesh were proven to be controlled by two genes, *wf* and a single recessive gene named *yf*, respectively (Kooistra, 1971; Whalen, 2005; Lu et al., 2015). The study on the color of cucumber flesh and skin will help to promote the breeding process of cucumber with a different color.

#### External Quality of Cucumber Fruits

Several genes and transcription factors are involved in the regulation of the external quality of cucumber fruits. Zhang C. et al. (2019) found that spine color was regulated by the *HEUKCHEEM* gene, mutations in *HEUKCHEEM* leading to a white spine in cucumber. Wang et al. (2015) revealed that *CsCER1* significantly influenced the biosynthesis of alkane so that further influenced the wax synthesis of cucumber, and *CsCER1* overexpression lines showed more wax crystallization phenotypes, whereas its RNA interference (RNAi) lines exhibited fewer wax crystallizations. Liu et al. (2018) analyzed 91 *NAC* gene homologs in cucumber and identified 13 *NAC* genes that can control fruit spine development. Yang et al. (2018) found that *CsMYB6* and *CsTRY* can negatively regulate the trichome initiation in cucumber and revealed that *CsMYB6* functions the upstream of *CsTRY* and that they can also form *CsMYB6*-*CsTRY* complex to function together in this progress. These results provide a reference for the cultivation of non-spiny and prickly cucumbers.

### Genetic Regulation of Flavor Quality in Cucumber

Research studies on flavor quality have become popular in recent years. Foods with good or special tastes will increase the pleasure in people and influence the digestion in people and absorption of nutrients (Beaulieu and Lea, 2006). The genes contributed to the flavor quality traits of cucumber fruits are shown again in Figure 1 and Table 2. It is confirmed that the degradation of linoleic acid and linolenic acid occurred rapidly after the disruption of cucumber tissues and gave rise to the flavor of fresh cucumber (Ligor and Buszewski, 2008).

**TABLE 2:** Genes related to the nutrient quality and flavor quality of cucumber fruits.

**Category**	**Traits**	**QTLs or Gene name**	**References**
Flavor quality	Aldehydes/alcohols	*9-CsHPL*	Liu, 2018
		*CsLOX2*	Wei, 2018
	Cucurbitacins	*Bt*, *Bt-2*, and *Bi*	Andeweg and Bruyn, 1959; Wehner et al., 2001; Gu et al., 2004; Shang et al., 2014
		*Csa008595*	Zhang et al., 2013
		*Csa6G088160*, *Csa6G088170*, and *Csa6G088180*	Zhou et al., 2016
	Catechins	WD40-repeat proteins, MYB, and bHLH	Xu et al., 2019a
		*CsPAL3*, *CsPAL5*, *CsC4H1*, *Cs4CL2*, *CsCHS2*, *CsCHI2*, *CsDFR2*, *CsF3H3*, and *CsANS*	Xu et al., 2019b
Nutrient quality	β-carotene	*ore*	Bo et al., 2012
		*CsZds*	Wang et al., 2012

#### The Scent of Cucumber Fruits

Aldehydes and alcohols are thought to mainly contribute to the fresh cucumber scent (Kemp et al., 1974; Hatanaka et al., 1975; Palma-Harris et al., 2001; Ligor and Buszewski, 2008; Hao et al., 2013). Forss et al. (1962) first isolated 2,6-nonadienal from cucumber. Subsequently, Schieberle et al. (1990) found that (*E,Z*)-2,6-nonadienal mainly caused the flavor of cucumbers with fresh cucumber odor and identified (*E*)-2-nonenal as the second important odor compound in cucumber. Since six-carbon (C6) and nine-carbon (C9) aldehydes play an important role in flavor during fruit development, changes in volatile substances in developing cucumber fruits were investigated in two *Cucumis sativus* L. lines (No. 26 and No. 14). C6 aldehyde content was higher during the early stages, whereas the C9 aldehyde content was higher during the latter stages in both lines (Chen et al., 2015). Lipoxygenase (LOX) and hydroperoxide lyase (HPL) are the two key pre-regulatory factors in the synthesis of cucumber aldehydes. Thereinto, the expression patterns of *9-CsHPL* are similar to the trend of C9, and the expression of *CsLOX2* is also significantly correlated with changes in C9 aldehyde aroma content (Liu, 2018; Wei, 2018). Subsequently, Wei et al. (2016) analyzed 85 volatile chemicals in 23 different tissues of cucumber and further found that TPS15 (encoded by *Csa3G040850*) mediated the biosynthesis of volatile terpenoid in the fruit tissues, which will promote future research studies on the physiological function of volatiles and improve the cucumber flavor breeding. Except for fresh cucumber odor, Yundaeng et al. (2015) also found a special pandan (*Pandanus amaryllifolius*), like fragrance in PK2011T202 (PKT) cucumber cultivar in Thailand. They further found that 2-acetyl-1-pyrroline (2AP) generated this fragrance, and a *betaine aldehyde dehydrogenase 2* (*BADH2*) mutant caused the biosynthesis of 2AP so that produced the pandan-like fragrance in PKT (Yundaeng et al., 2015). Two cucumber accessions, PKT and 301,176 (301), an inbred line from Clover Seed Company, Hong Kong, possessing no fragrance, were used to determine the mode of inheritance of these recessive fragrance traits and each controlled by a specific gene (Pramnoi et al., 2013).

#### The Bitterness of Cucumber Fruits

The bitterness of cucumber fruits is also of great popularity in the research study of flavor quality traits. Research studies have demonstrated that the Cucurbitacins (Ct) caused the bitterness in cucumber fruits (Rice et al., 1981; Balkema-Boomstra et al., 2003). Since the production of bitterness will lead to fatal losses in the sale of cucumbers, cultivating varieties of cucumbers without bitterness is of great significance for improving the efficiency of cucumber sales. Genetic mechanism of cucumber bitterness showed great complexity. Wehner et al. (2001) found that two dominant genes, Bt (bitter fruit) and Bt-2 (bitter fruit-2), controlled the bitterness of cucumber. In addition, bi (bitter-free cotyledons) gene and the fruit bitterness need both Bi and Bt genes (Andeweg and Bruyn, 1959; Gu et al., 2004; Shang et al., 2014). Further research studies by Zhang et al. (2013) proved that the candidate gene of bi-1 is considered to be the terpene synthase gene named *Csa008595*. Besides, Shang et al. (2014) further demonstrated that Bt can regulate the biosynthesis of cucurbitacin C (CuC) in the cucumber fruits and identified 11 cucumber bitterness biosynthesis, regulation, and domestication-related genes. In addition, the biosynthetic pathway and main regulators of cucurbitacin in cucumber have also been identified. By applying a comparative genomic study, Zhou et al., reported that the independent mutations of the homologous transcription factor genes in the three cucurbits may lead to a significant reduction in fruit bitterness, which may be the reason for the convergence and domestication of bitter wild cucurbits. A syntenic gene cluster that regulates both the tissue-specific biosynthesis of cucurbitacin and the loss of bitter phenotypes associated with the fusion and domestication of wild cucurbits has also been reported in this study. They also found that *Csa6G088160*, *Csa6G088170*, and *Csa6G088180* in cucumber can participate in the biosynthesis of cucurbitacin C (Zhou et al., 2016).

In addition to cucurbitacin, catechins are also one of the key factors that cause cucumber to produce astringency. Xu et al. (2019a) found that tryptophan–aspartate acid (WD40)-repeat protein, avian myeloblastosis viral oncogene homolog (MYB), and basic helix–loop–helix (bHLH) also play an important role in the biosynthesis of catechins. They further found that some genes related to phenylalanine ammonia lyase (PAL)-*CsPAL3* and *CsPAL5*, to cinnamate 4-hydroxylase (C4H)-*CsC4H1*, to 4-coumarate-CoA ligase (4CL)-*Cs4CL2*, to chalcone synthase (CHS)-*CsCHS2*, to chalcone isomerase (CHI)-*CsCHI2*, to flavanone 3-hydroxylase (F3H)-*CsF3H3*, to dihydroflavonol 4-reductase (DFR)-*CsDFR2*, and to anthocyanidin synthase (ANS)-*CsANS* are important regulators of catechin biosynthesis in cucumber fruits (Xu et al., 2019b). But there is currently no clear evidence on how catechins are regulated.

### Genetic Regulation of Nutrient Quality in Cucumber

Qiao et al. (2005) reported that crude protein, Vitamin C (VC), soluble reductive sugar, soluble solids, and moisture were five important nutrient components in cucumber. Meanwhile, soluble solids can directly affect nutritional quality, with the greatest impact, while VC, soluble reducing sugars, and crude protein indirectly affect nutritional quality through soluble solids (Qiao et al., 2005). The genes related to the nutrient quality traits of cucumber fruits are shown in Figure 1 and Table 2. The heritability of the soluble sugar content of cucumber is higher, and the selection of early generation has a better effect on it (Xu et al., 2001). Zhang et al. (2020) found that the relationship between soluble sugar and water is positively correlated, while ascorbic acid is negatively correlated with water and soluble sugar. Besides, the contents of β-carotene, a provitamin A, were also important nutrient qualities in cucumber, its contents in cucumber were regulated by an *ore* gene, and seven simple sequence repeat (SSR) markers were identified linking to the locus controlling β-carotene quantity (Bo et al., 2012). On this basis, Wang et al. (2012) used RACE technology to successfully clone the complementary DNA (cDNA) sequence of the ζ-carotene dehydrogenase (ZDS) gene (*CsZds*) and speculated that the gene may be related to the accumulation of β-carotene in cucumber fruits. However, there existed fewer genetic research studies on nutrient qualities in cucumber even until now, and further studies are needed to identify factors related to nutrient qualities formation in cucumber.

## Physiological Regulation of Fruit Quality in Cucumber

### Physiological Regulation of Commercial Quality in Cucumber

**FIGURE 2:**
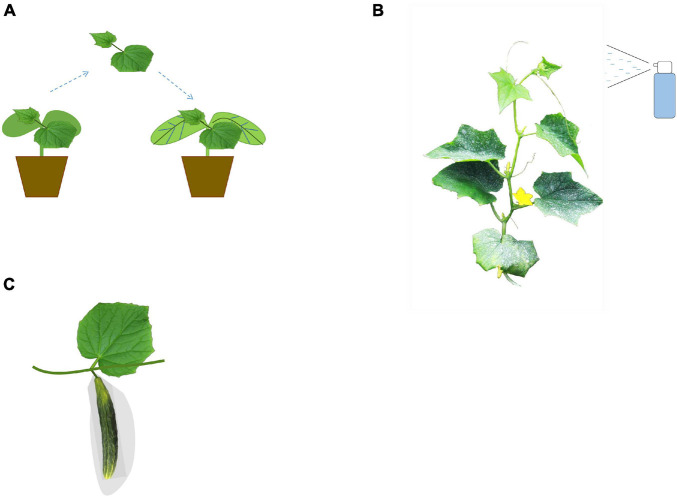
Illustration of the cultivation techniques in cucumber fruit quality breeding. Grafting technique **(A)**, application of exogenous fertilizers and growth regulators **(B)**, and bagging technology **(C)** are widely used in cucumber fruit quality breeding.

Cucumber plants usually have poorly developed root systems, rending them vulnerable to infection by various pests and diseases; thus, the cucumber growers have to apply various techniques to improve the commercial quality of cucumber fruits (Figure 2). Grafting was wildly used to improve the stress resistance in cucumber, which could also generate positive influences on improvement in their fruit commercial quality (King et al., 2008; Colla et al., 2013). Grafting can also influence the transcript expression levels in cucumber. For example, Zhang J. et al. (2019) found that compared to those self-rooted cucumber, the grafted cucumber showed a higher expression level of the Apetala2/ethylene-responsive factor (AP2/ERF)-type transcription factor *CsWIN1*, and *CsWIN1* further promoted the expression of several key wax biosynthesis and transporter genes so that reflected a glossier appearance. Besides, the usage of plant growth regulators is also an effective measure to alter the fruit qualities in cucumber. Qian et al. (2018) found that N-(2-chloro-4-pyridyl)-N′-phenylurea (CPPU) treatment produced a positive effect on cucumber appearance for the increased flesh firmness. However, gibberellin A4 + A7 (GA4 + 7) treatment reduced its commercial quality (Qian et al., 2018). Hypoxia treatment can inhibit the fresh weight of cucumber fruit. Under hypoxia stress, increasing the amount of exogenous calcium can increase the fresh weight of the fruit (He et al., 2018).

### Physiological Regulation of Flavor Quality in Cucumber

Grafting not only affects the commercial quality of cucumbers but also affects the total amount of aroma substances and characteristic esters in the fruit (Pérez et al., 2002). Dong et al. (2013) found that the increased content of volatile substances such as acetaldehyde in the fruits of cucumbers grafted to pumpkins led to a decrease in the flavor and taste of the fruits. Zhao et al., found that compared with those self-grafted cucumbers, the total content of alcohols, aldehydes, olefins, and acids increased of those cucumber plants that were grafted onto “Weisheng NO.1” (*Cucurbita moschata* hybrids) rootstock, so the flavor quality was significantly improved, while the cucumber plants grafted onto *Cucurbita ficifolia* rootstock showed the opposite trend. Grafting of different rootstocks will also significantly affect the expression of genes related to glycolysis, fructose metabolism, and α-linolenic acid metabolism in the scion, thereby changing the flavor quality of grafted cucumbers (Zhao et al., 2018). Different grafting methods will also affect the flavor of cucumber fruits (Lee, 1994). Peng et al. (2010) comprehensively evaluated the sweetness, bitterness, astringency, moisture, and other aspects of cucumber fruit and found that the taste quality of cucumber after double-cut root grafting was the best, while the taste quality of cucumber after double-root grafting was poor. Wang et al. (2019) found that compared with conventional grafting and self-rooting seedlings, the method of interstock grafting can significantly increase the types and contents of volatile substances in cucumber fruits. Besides, bagging was also an effective measure to improve the fruit quality. Shan et al. (2020) demonstrated that cucumber fruits were found to have enhanced fruit flavor quality after bagging, for the elevated relative proportion of C6 aldehyde, (*E,Z*)-2,6-nonadienal/(*E*)-2-nonenal ratio, and linoleic/α-linolenic acid ratio.

### Physiological Regulation of Nutrient Quality in Cucumber

Grafting also has an influence on nutrient qualities in cucumber fruits. For example, in the study of Zhao et al. (2018) when cucumber was grafted onto “Weisheng NO.1,” the soluble solid content in the fruit was significantly higher than that of the self-grafted group. Besides, the contents of the sugar, organic acids, amino acids, and alcohols were greatly increased when grafting “xintaimici” cucumber on the “GNo.45” pumpkin (*Cucurbita moschata*) (Miao et al., 2019). Dong et al. (2018) also found that elevated carbon dioxide (CO_2_) concentration and high nitrogen (N) application can also increase the content of nutrients such as fructose and glucose in cucumber by promoting the carbon translocation from source leaves to fruits. Qian et al. (2018) also found that gibberellin GA4 + 7 treatment improved the nutrient quality in cucumber fruits but decreased its commercial quality. On the contrary, CPPU treatment had a negative effect on the nutritional quality of cucumber (Qian et al., 2018). Moreover, He et al. (2018) found that exogenous calcium treatment on cucumbers under hypoxic stress results in an increased soluble sugar content in cucumber fruits so that enhances its nutrient quality. Ali et al. (2019) found that cucumber growth can be improved by adding arbuscular mycorrhizal strain (AM: *Glomus versiforme* L.) inoculant with organic substrates (GS), and GS + AMF (arbuscular mycorrhizal fungi) treatments increase the total soluble solids of cucumber fruits and soluble sugar content, thereby improving the nutrient qualities of cucumber fruits. With the rapid development of facility cultivation technology, cucumber has now become one of the main crops cultivated in protected areas. Di-n-butyl phthalate (DBP) is widely used as a plasticizer in plastic films because it can increase the toughness and elasticity of products. However, one of its main components, dibromophenol, can cause agricultural pollution that leads to food safety problems, and it has been widely concerned (Heudorf et al., 2007). DBP stress has also a detrimental effect on the contents of organic acids, vitamin C, soluble protein, and soluble sugar in cucumber fruits and resulted in the residue of dibromophenol under protected cultivation conditions. Although the residual dibromophenol in cucumber fruits is below the risk threshold, the potential health risks cannot be ignored (Wang et al., 2016). Therefore, in the process of studying cucumbers, attention should also be paid to the safety of the protected cultivation of cucumbers.

## Perspective

Compared to the traditional model plants such as *Arabidopsis* and rice, cucumber has some advantages as a new model plant for studies on gene function during fruit development. However, the fruit development of cucumber is a complex biological process, which is affected both by internal genes and external environmental factors. We still need firstly a comprehensive understanding of cucumber quality traits and problems of cultivation skills such as the production of grafted rootstocks, genetic deterioration in breeding, and soil renovation under protected cultivation. Then, we should analyze the compositions of good-quality fruits, such as the types and content of flavor substances, the nutrients, and pigments in cucumber fruits by using high-throughput, high-resolution, and high-sensitivity modern instrumental analysis methods. Combining with the taste evaluation system of consumers, the breeding goal of cucumber flavor quality can be established and then the genetic mechanism of formation of cucumber fruit quality can be explored. Molecular breeding techniques and methods are used to create new varieties with the best commercial quality (the top flower with thorns and straight strips), nutritional quality (rich in nutrients), and flavor quality (clear fragrance and no bitterness) to meet consumer demand eventually.

In recent years, biotechnologies such as fine mapping, cloning, and transgenesis of genes for important cucumber fruit traits have developed rapidly, and molecular markers combined with traditional breeding methods have been widely used. We will accelerate the completion of the improvement of fruit quality and achieve the breeding goal of high quality, high yield, and stable yield in cucumber fruit production.

## Author Contributions

CY, JZ, JY, and HW conceived the review, conducted the literature review, and wrote the manuscript. CW and TL collected the literature. SF provided critical comments on the manuscript. All authors read and approved the manuscript.

## Conflict of Interest

The authors declare that the research was conducted in the absence of any commercial or financial relationships that could be construed as a potential conflict of interest.

## Publisher’s Note

All claims expressed in this article are solely those of the authors and do not necessarily represent those of their affiliated organizations, or those of the publisher, the editors and the reviewers. Any product that may be evaluated in this article, or claim that may be made by its manufacturer, is not guaranteed or endorsed by the publisher.
